# Supplementary material for: Differences in the perception of harm assessment among nurses in the patient safety classification system
Source: PLoS One. 2020 Dec 7;15(12):e0243583. doi: 10.1371/journal.pone.0243583 (PMC7721130; doi:10.1371/journal.pone.0243583)
Supplement: S1 Questionnaire — (PDF) [file pone.0243583.s001.pdf]

All contents investigated in this survey are only for statistical purposes and are protected by the Statistical Law (Articles 33 and 34).

ID			

Differences in the perception of harm assessment among nurses in the patient safety classification system

Hello,

This survey is to investigate differences in perception of harm assessment among nurses in the patient safety classification system.

Your sincere answers will be used as important data for the improvement and development of the patient safety classification system in the future, and will become a valuable foundation for preparing a system for patient safety in the clinical field, and will not be used for any other purpose.

You have the right not to participate in this survey and there will be no penalty for not participating in the study. You can also stop participating in the study by your own doctor, even while the study is in progress.

Respondents' data will be used only as a research purposes, and respondents' information is thoroughly protected in accordance with Article 33 of the Statistical Law, so we ask that you respond faithfully to all questions so that accurate data can be collected.

Thank you for participating in this study.

If you have any questions while filling out the questionnaire, please contact the principal investigator below.

December, 2017

■ Principal investigator : Prof. Eunhee Shin, Department of Nursing sciences,
Sangji University (HP: 010-6278-9622)
Kwangmi Lee, Department of Nursing,
National Cancer Center (HP: 010-7624-1111)

Consent form

I fully understand the purpose of this study, and I understand and agree to the use of the data I have responded to. I also confirm that I have been informed that I can withdraw my consent at any time if I wish.

Agree □ Not agree □

Name: (Signature)

*** We provided separate explanations including the purpose of the study and the study procedure, etc and consent forms, to the study subjects.*

※ Before we begin, please tell us about yourself and your experiences of patient safety

1. Gender?

____① Male ____② Female

2. Current department?

____① General ward ____② Special ward (ICU, ER, etc)

____③ Outpatient department ____④ Others ( )

3. Working period at current job (year)?

____① <1 ____② ≥1-<5

____③ ≥5-<10 ____④ ≥10

4. Job satisfaction at current job?

____① Very satisfied ____② Satisfied

____③ Unsatisfied ____④ Very unsatisfied

5. Whether or not getting a patient safety training at current job?

____① Yes ____② No

6. Method of a patient safety training? (duplication check)

____① Theoretical lectures ____② Case-based discussion training

____③ Certificated brochure training ____④ Department conveying training

____⑤ Others ( )

7. Contents of a patient safety training? (duplication check)

____① Understanding Patient Safety ____② Time and method of patient identification

____③ Grade and criteria of patient safety incident reporting ____④ Incident reporting procedures

____⑤ Inpatient care management ____⑥ Activating near miss reporting

____⑦ Others ( )

7. Time of a patient safety training? (hour)

____① <1 ____② ≥1-<4

____③ ≥4-<8 ____④ ≥8

9. Whether or not participating events(Special lecture, seminar, Campaign, etc) of a patient safety at current job?

____① Yes ____② No

10. Experience of patient safety accident at current job?

____① Yes → Go to questions 11, 12, 13

____② No

11. Type of patient safety accident? (duplication check)

____① Near miss

____② No harm safety event

____③ Mild/Moderate/Severe safety event

____④ Sentinel event

12. Reporting on patient safety accidents?

____① Yes

____② No

13. Types of patient safety accidents experienced? (duplication check)

____① Surgery

____② Delivery

____③ Treatment procedure

____④ Anesthesia

____⑤ Clinical examination

____⑥ Blood transfusion

____⑦ Medication

____⑧ Infection

____⑨ Computerized disorder

____⑩ Medical equipment/Medical device

____⑪ Hospital meal

____⑫ Fall

____⑬ Treatment material contamination /failure

____⑭ Suicide/Self-harm

____⑮ Others (   )

※ Harm Score

Score for Harm	Patient outcome
Near miss (A)	An circumstances or events that have the capacity to cause error, such as disorganized medical equipment
Near miss (B)	An error occurred but the error did not reach the patient
No harm (C)	An error occurred that reach the patient but did not cause patient harm
Mild harm (D)	An error occurred that reached the patient and required monitoring to confirm that it resulted in no harm to the patient and/or required intervention to preclude harm
Moderate harm (E)	An error occurred that may have contributed to or resulted in temporary harm to the patient and required intervention
Severe harm (F)	An error occurred that may have contributed to or resulted in permanent patient harm and required the short-term or long-term hospitalization
Sentinel Event	An error occurred that may have contributed to or resulted in the patient's death

※ Harm Duration

Score for duration	
Permanent	An error occurred to the patient persisting over a year
Temporary	An error occurred to the patient persisting less than a year

※ The following is a case of patient safety incident hat occurred in the clinical field. Please read scenario and fill out each question.

[Scenario 1]
A 61-year-old female patient was hospitalized for cervical cancer for hysterectomy. As a result of regular examination, VDRL (Venereal Research Laboratory) reactive and TPHA (Treponema Pallidum Hemagglutination Assay) reactive were confirmed. The physician prescribed an additional injection of Benzathine penicillin G 2.4 million IU muscles. While charge nurse A was not aware of the additional prescription, nurse B received a phone call from the physician asking whether or not to perform the Benzathine penicillin skin test at around 11 am, and informed them that the drug has not yet been delivered to the ward and it has not been administered. Nurse B, who confirmed that the drug had arrived at around 12 o'clock, performed a Benzathine penicillin skin test to help the nurse A who seems busy, and confirmed that it was negative and informed the nurse A. At around 1 pm, nurse B received a call from the doctor asking whether or not to take the medication, and confirmed that the nurse in charge of A was still busy, and Benzathine penicillin G was in the preparation room and informed that she would take it immediately. The physician in charge is urging to take the medication as soon as possible, then the nurse B who judged that the medication would not be administered immediately because charge nurse A was too busy, mixed 50cc of serile normal saline with benzathine phenicillin G 2.4 million IU and injected intravenously for about 60 minutes without accurately confirming the prescription.

1.1 Please mark the harm scale of the above patient safety scenario as determined by you.

____① Near miss (A) ____② Near miss (B) ____③ No harm (C) ____④ Mild harm (D)

____⑤ Moderate harm (E) ____⑥ Severe harm (F) ____⑦ Sentinel Event

1.2 Please mark the harm duration of the above patient safety scenario as determined by you.

____① Permanent ____② Temporary ____③ Unknown

[Scenario 2]
Patient A had surgery to pin a pin into a bone due to a fracture, and over time, patient A underwent a pin removal operation. After the pin removal, patient A's left foot was swollen and numb, and pain in his left ankle persisted. Therefore, patient A and his family asked the hospital about the symptoms, but the doctor in charge of the hospital said the surgery went well and discharged patient A. After discharge, Mr. A's left foot is constantly swollen and numb, and he was hospitalized again due to pain in his left ankle, which was stabbing with a needle, and after treatment, he was informed that the peroneal nerve broke due to negligence in the process of pinning a bone with a fracture and the nerves reconnected.

2.1 Please mark the harm scale of the above patient safety scenario as determined by you.

____① Near miss (A) ____② Near miss (B) ____③ No harm (C) ____④ Mild harm (D)

____⑤ Moderate harm (E) ____⑥ Severe harm (F) ____⑦ Sentinel Event

2.2 Please mark the harm duration of the above patient safety scenario as determined by you.

____① Permanent ____② Temporary ____③ Unknown

[Scenario 3]

Patient A complained of dyspnea and swelling from 20 days before hospitalization and performed chest X-ray examination, etc., After examination, he was diagnosed with chronic heart failure (due to dilated cardiomyopathy), atrial fibrillation, high blood pressure, anemia, etc. While the symptoms such as dyspnea were improved by progressing the treatment, he complained of chest tightness with arrhythmia, but the symptoms improved. Before discharge, the medical staff explained that CT angiography was necessary to evaluate the patient's potential for exacerbation of pulmonary embolism or heart disease, and CT angiography was performed after obtaining consent from the patient. After CT angiography, the patient complained of tightness in the chest, and from the next day he began to show symptoms such as nausea, vomiting, and diarrhea, and then continued to lie down and sleep. Therefore, the medical staff diagnosed the patient's condition as acute kidney injury due to contrast media, hyponatremia, and hyperkalemia and treated them.

3.1 Please mark the harm scale of the above patient safety scenario as determined by you.

____① Near miss (A) ____② Near miss (B) ____③ No harm (C) ____④ Mild harm (D)

____⑤ Moderate harm (E) ____⑥ Severe harm (F) ____⑦ Sentinel Event

3.2 Please mark the harm duration of the above patient safety scenario as determined by you.

____① Permanent ____② Temporary ____③ Unknown

[Scenario 4]

An 80-year-old male patient underwent an open surgery due to bleeding in bladder neck and prostate gland while undergoing Holeb surgery. After the operation, the patient complained of severe abdominal pain, and his heart rate rose more than 140/m, and underwent abdominal pelvic computerized tomography under general surgery cooperation, and as a result, he underwent a re-examination of the small intestine, which was perforated by peritonitis and bilaterally pleural fluid findings. Later, when patients showed septic symptoms such as high fever, blood culture tests were conducted, and as a result, Staphylococcus Epidermidis, one of the multidrug-resistant bacteria, was detected and Vancomycin was administered. When the patient showed symptoms of thrombosis after the administration of Vancomycin, the medical team suspected acute renal failure resulting from the administration of Vancomycin through cooperation with kidney internal medicine, stopped the administration of Vancomycin, and performed continuous renal replacement therapy (CRRT). However, as the patient's blood stool continued, intestinal endoscopy and upper gastrointestinal endoscopy were performed, the second part of the operation was suspected of re-bleeding the small intestine, and the operation was performed to resect part of the small intestine. After the third surgery, the patient's oxygen saturation decreased by 87%, and the medical team performed an endotracheal intubation, and Staphylococcus and candida were detected in the blood culture bacteria test, and Teicoplanin and Meropenem antibiotics were administered.

4.1 Please mark the harm scale of the above patient safety scenario as determined by you.

____① Near miss (A) ____② Near miss (B) ____③ No harm (C) ____④ Mild harm (D)

____⑤ Moderate harm (E) ____⑥ Severe harm (F) ____⑦ Sentinel Event

4.2 Please mark the harm duration of the above patient safety scenario as determined by you.

____① Permanent ____② Temporary ____③ Unknown

[Scenario 5]
In order to carry out the test prescription all at once in a busy situation, blood was collected for transfusion of patient A but blood was put into the sample container of patient B, which had been labeled in advance, so blood was prepared for patient B. Patient B's blood was transfused due to a blood prescription for patient A. Patient A immediately complained that his chest was stuffy when the transfusion began, and immediately stopped the transfusion and checked that the sample had changed.

5.1 Please mark the harm scale of the above patient safety scenario as determined by you.

____① Near miss (A) ____② Near miss (B) ____③ No harm (C) ____④ Mild harm (D)

____⑤ Moderate harm (E) ____⑥ Severe harm (F) ____⑦ Sentinel Event

5.2 Please mark the harm duration of the above patient safety scenario as determined by you.

____① Permanent ____② Temporary ____③ Unknown

[Scenario 6]
The patient underwent surgery to restore the intervertebral disc between the 4th and 5th vertebrae in the hospital due to lumbar herniated intervertebral disc, which is a symptom of compression of the spinal nerve. At the time of the operation, the medical team performed an operation to incisional of intervertebral disc after attaching the medical clip to the spine No. 4 and 5, but later discovered that the actual spinal cord with the medical clip was the spinal cord No. 3 and 4, not the spine No. 4 and 5, and the medical team took care of the situation and completed the operation. After the operation, the patient began to occur purulent inflammation in the intervertebral disc No. 3, 4, 5, and the first and fifth lumbar herniated intervertebral disc resulted in the compression of the vertebrae, which eventually led to sexual dysfunction.

6.1 Please mark the harm scale of the above patient safety scenario as determined by you.

____① Near miss (A) ____② Near miss (B) ____③ No harm (C) ____④ Mild harm (D)

____⑤ Moderate harm (E) ____⑥ Severe harm (F) ____⑦ Sentinel Event

6.2 Please mark the harm duration of the above patient safety scenario as determined by you.

____① Permanent ____② Temporary ____③ Unknown

[Scenario 7]
The patient underwent a left lung lobotomy for lung cancer diagnosis and came to the ward with a chest tube inserted from the operating room. On the third day after the surgery, the charge nurse saw that the patient's chest tube drainage bottle was filled with a lot of fluid during night shift and decided to exchange the drainage bottle. The nurse who went to the patient by preparing a new chest tube drainage bottle and Kelly to clamp, locked the patient's chest tube with Kelly for the exchange of the drain bottle and separated the connection between the drain bottle and the chest tube. At that moment, Kelly, which had been loosely locked as the patient moved, was released and the patient complained of discomfort as he breathed in. The nurse didn't think Kelly would be unlocked because the lock had never been unlocked. The nurse connected the chest tube to the new drainage bottle and made the patient cough. After that the nurse reported it to the doctor. When the patient first coughed, air was seen coming out of the chest tube but no more came out. The chest X-ray examination was performed immediately and there were no problems.

7.1 Please mark the harm scale of the above patient safety scenario as determined by you.

____① Near miss (A) ____② Near miss (B) ____③ No harm (C) ____④ Mild harm (D)

____⑤ Moderate harm (E) ____⑥ Severe harm (F) ____⑦ Sentinel Event

7.2 Please mark the harm duration of the above patient safety scenario as determined by you.

____① Permanent ____② Temporary ____③ Unknown

[Scenario 8]
The nurse diluted 50mg of isoket into the fluid at around 22:50 to inject the 34-year-old male patient, who visited the emergency room for chest pain and as the main hospital and started injection with 10cc/hr using the infusion pump. At around 23:35 after premeditation, the patient went to the CT room for a 3D CT examination and then transfered to the general ward at around 23:55. After hospitalization, the charge nurse found that the solution diluted with 50mg of isoket was fully dropped and 150cc was injected then immediately reported to the physician. The staff in the CT room said that they could not remember whether the infusion pump was locked or not after injecting contrast with the infusion pump removal for CT scans, and the person who transfered the patient said that the patient was transfered just as the patient came out of the CT room.

8.1 Please mark the harm scale of the above patient safety scenario as determined by you.

____① Near miss (A) ____② Near miss (B) ____③ No harm (C) ____④ Mild harm (D)

____⑤ Moderate harm (E) ____⑥ Severe harm (F) ____⑦ Sentinel Event

8.2 Please mark the harm duration of the above patient safety scenario as determined by you.

____① Permanent ____② Temporary ____③ Unknown

[Scenario 9]
Following reports of Yeast's growth in blood culture in patients with idiopathic pulmonary fibrosis, the physician decided to use Amphotericin B and for safety, the physician prescribed a test dose of 1 mg, 24 mg 4 hours later, 25 mg 12 hours later again, and then 50 mg daily. The nurse A had no experience of actually administering Amphotericin B, so she scheduled to take 1 mg of test dose at 1 p.m., 24 mg at 5 p.m., and 25 mg at 9 a.m. the next day after 16 hours as of 5 p.m., according to the prescriptions and handed over it to the evening shift nurse. Night shift nurse B, who was handed over to the evening shift nurse, had been ordered to administer Amphotericin B 25 mg at 5 a.m. the next day, but she thought it was a mistake in the time calculation of physician and handed it over to the day shift nurse to administer the medicine at 9 a.m. The day shift nurse checked with physician before taking the medication at 9 a.m. and found that the 16-hour criterion was found to be 1 p.m. when the test dose was administered.

9.1 Please mark the harm scale of the above patient safety scenario as determined by you.

____① Near miss (A) ____② Near miss (B) ____③ No harm (C) ____④ Mild harm (D)

____⑤ Moderate harm (E) ____⑥ Severe harm (F) ____⑦ Sentinel Event

9.2 Please mark the harm duration of the above patient safety scenario as determined by you.

____① Permanent ____② Temporary ____③ Unknown

♣ Thank you very much for your response!! ♣
