# Supplementary material for: Differences in the perception of harm assessment among nurses in the patient safety classification system
Source: PLoS One. 2020 Dec 7;15(12):e0243583. doi: 10.1371/journal.pone.0243583 (PMC7721130; doi:10.1371/journal.pone.0243583)
Supplement: S2 Questionnaire — (PDF) [file pone.0243583.s002.pdf]

이 조사에 조사된 모든 내용은 통계목적 이외에는
절대로 사용할 수 없으며 그 비밀이 보호되도록
통계법(제33조 및 제34조)에 규정되어 있습니다.

ID			

환자안전 분류체계에서 간호사의 위험도(harm) 사정에 대한 인식 차이를 위한 설문조사

안녕하십니까?

본 설문조사는 환자안전 분류체계에서 간호사 간의 위험도(harm) 사정에 대한 인식 차이를 조사하기 위한 설문조사입니다.

귀하의 성의 있는 답변은 향후 환자안전 분류체계의 개선과 발전을 위한 중요한 자료로 사용되어 임상현장에서 환자안전을 위한 시스템 마련에 귀중한 밑거름이 될 것이며, 그 이외의 용도로는 사용되지 않을 것입니다.

귀하는 본 설문조사에 참여하지 않을 권리가 있으며 연구에 참여하지 않아도 불이익이 발생하지 않습니다. 또한 연구가 진행되는 도중이라도 본인의 의사에 의해 연구 참여를 중단할 수 있습니다.

응답하신 내용은 연구목적을 위한 참고자료로만 활용되며, 통계법33조에 의거 응답자의 정보는 철저히 보호되오니, 정확한 자료가 수집될 수 있도록 모든 문항에 대하여 빠짐없이 성실하게 답변해 주시기를 부탁드립니다.

바쁘신 가운데 참여해 주셔서 감사합니다.

설문지를 작성하시는 중에 의문점이 있으시면 아래 연구책임자에게 문의바랍니다.

2017 년 12 월

■ 연구책임자 : 상지대학교 간호학과 신 은희 교수 (연락처 : 010-6278-9622)
적정진료관리팀 이 광미 팀장(연락처 : 010-7624-1111)

연구 동의서

본인은 본 연구의 목적에 대해 충분히 이해하였으며 본인이 응답한 자료의 활용에 대하여 이해하고 동의합니다. 또한 동의를 철회하고자 할 때 언제든지 이를 행사할 수 있다는 사실을 전달 받았음을 확인합니다.

동의 □ 비동의 □

성명: (서명)

※ 다음은 귀하의 **일반적 사항**을 묻는 질문입니다. 각 문항에 대해 작성하여 주시기 바랍니다.

1. 귀하의 성별은?
____① 남 ____② 여

2. 귀하의 현재 부서는?
____① 일반병동(이식실 포함) ____② 특수병동(중환자실, 응급실, 회복실 등)
____③ 외래 ____④ 기타 부서( )

3. 귀하의 현재 직장에서 근무한 기간은?
____① 1년 미만 ____② 1년 이상 5년 미만
____③ 5년 이상 10년 미만 ____④ 10년 이상

4. 귀하의 현재 직장에서의 직무에 대해 만족하십니까?
____① 매우 만족한다 ____② 만족한다
____③ 불만족이다 ____④ 매우 불만족이다

5. 귀하는 현재 근무하고 있는 기관에서 환자안전에 대한 교육을 받았습니까?
____① 예 ____② 아니오

6. 귀하의 환자안전 교육 방법은 다음 중 어디에 해당합니까? (중복 답변 가능)
____① 이론 강의 ____② 사례기반 토론 교육
____③ 인증 책자 교육 ____④ 부서 전달교육
____⑤ 기타 ( )

7. 귀하께서 환자안전에 대해 교육 받은 내용은 다음 중 어디에 해당합니까? (중복 답변 가능)
____① 환자안전의 이해 ____② 환자확인 시점과 방법
____③ 환자안전 사고보고 등급과 기준 ____④ 사고보고 절차
____⑤ 입원환자 병문안 관리 ____⑥ 근접오류 보고 활성화
____⑦ 기타( )

7. 귀하의 환자안전 교육 시간은 다음 중 어디에 해당합니까?
____① 1시간 미만 ____② 1시간 이상 4시간 미만
____③ 4시간 이상 8시간 미만 ____④ 8시간 이상

9. 귀하는 근무하고 있는 기관에서 환자안전 주간행사(특강, 세미나, 캠페인 등)에 참여한 적이 있습니까?
____① 예 ____② 아니오

10. 귀하는 현재 기관에서 근무하면서 환자안전 사고를 경험한 적이 있습니까?

____① 예 → 11,12,13번 문항으로 ____② 아니오

11. 경험한 환자안전 사고는 다음 중 어디에 해당합니까? (중복 답변 가능)

____① 근접오류 ____② 무해사건

____③ 위해사건 ____④ 적신호사건

12. 경험한 환자안전 사고에 대해서 보고서를 작성하였습니까?

____① 예 ____② 아니오

13. 귀하께서 경험한 환자안전 사고는 다음 중 어느 것에 해당합니까?
(해당되는 모든 사항에 답해주시길 바랍니다)

____① 수술 ____② 분만

____③ 처치. 시술 ____④ 마취

____⑤ 검사 ____⑥ 수혈

____⑦ 투약 ____⑧ 감염

____⑨ 전산장애 ____⑩ 의료장비/기구

____⑪ 식사 ____⑫ 낙상

____⑬ 진료재료 오염/불량 ____⑭ 환자의 자살/자해

____⑮ 기타( )

※ 위험도 분류(Harm Score)

Code	정의
근접오류(A)	정돈되지 않은 의료장비와 같이 위해사건을 일으킬 수 있는 환경
근접오류(B)	사고가 발생은 하였으나 환자에게 도달되지 않음
무해사건(C)	사고가 발생하였으나 환자에게 위해가 발생하지 않았고 추가적인 모니터링도 필요하지 않음
무해사건(D)	사고가 발생하여 환자에게 위해가 발생하지 않을지 또는 위해를 예방하기 위해 추가적인 중재가 필요한 경우
위해사건(E)	사고가 발생하여 환자에게 일시적인 위해가 생겨 추가적인 치료 또는 중재가 필요한 경우
위해사건(F)	사고가 발생하여 환자에게 일시적인 위해가 생겨 단기 또는 장기간의 입원 치료가 필요한 경우
적신호사건	사고가 발생하여 환자에게 사망에 근접한 상태를 야기하거나 영구적인 위해를 끼친 경우 또는 환자가 사망하거나 사건이 환자 사망에 기여한 경우

※ 위험도 기간(Harm Duration)

분류	정의
**영구적(Permanent)**	사고가 발생하여 환자에게 일시적인 위해가 생겨 1년 이상 지속될 경우
**일시적(Temporary)**	사고가 발생하여 환자에게 일시적인 위해가 생겨 1년 미만으로 지속될 경우

※ 다음은 임상현장에서 발생하였던 환자안전 사례입니다. 사례를 보시고, 각 문항에 대해 작성하여 주시기 바랍니다.

[사례 1]

61세 여자 환자는 자궁경부암으로 자궁적출술을 받기 위해 입원하였으며, 정규 검사 결과 VDRL(Venereal Research Laboratory) reactive, TPHA(Treponema Pallidum Hemagglutination Assay) reactive가 확인되어 주치의는 Benzathine penicillin G 240만 IU 근육주사를 추가 처방하였다. A담당간호사가 추가 처방을 모르고 있는 상태에서 B간호사는 오전 11시경 Benzathine penicillin skin test의 시행여부를 묻는 주치의의 전화를 받고 확인하여 아직 약이 병동에 오지 않아 시행하지 못했다고 알려주었다. 12시경 약이 도착한 것을 확인한 B간호사는 바빠 보이는 A담당간호사를 도와주기 위해 자신이 Benzathine penicillin skin test를 시행하여 negative인 것을 확인하고 A담당간호사에게 알려주었다. 오후 1시경 B간호사는 투약 여부를 묻는 주의의의 전화를 다시 받고 확인해보니 A담당간호사는 여전히 바빠보였고, Benzathine penicillin G는 준비실에 그대로 있어 바로 투약하겠다고 알렸다. 빨리 투약해달라는 담당 주치의의 재촉이 있는데 담당간호사는 바빠 보여 바로 투약이 실시되지 않을 것 같다고 판단한 B간호사는 처방을 정확하게 확인하지 않은 채 생리식염수 50cc에 Benzathine penicillin G 240만 IU를 혼합하여 약 60분간 정맥주사하였다.

1.1 귀하가 판단하는 상기 환자안전 사례의 위험도에 √표시를 해주시기 바랍니다.

____① 근접오류(A) ____② 근접오류(B) ____③ 무해사건(C) ____④ 무해사건(D)

____⑤ 위해사건(E) ____⑥ 위해사건(F) ____⑦ 적신호사건

1.2 귀하가 판단하는 상기 환자안전 사례의 위험도 기간에 √표시를 해주시기 바랍니다.

____① **영구적(Permanent)** ____② **일시적(Temporary)** ____③ 알 수 없음(Unknown)

[사례 2]

A씨는 골절로 인해 뼈에 핀을 박는 수술을 하였고, 시간이 흘러 A씨는 핀 제거술을 받았다. 핀 제거술이 끝나고 A씨는 좌측 발이 붓고 감각이 없어졌으며, 또한 좌측 발목에 통증이 지속적으로 전해졌다. 이에 A씨와 가족들은 이러한 증상에 대해 병원에 문의를 하였지만 병원의 담당 의사는 수술은 잘 되었다고만 대답하고 A씨를 퇴원시켰다. 퇴원 후, A씨는 좌측 발이 지속적으로 붓고 무감각하며, 좌측 발목이 바늘로 찌르는 듯한 통증으로 잠을 이루지 못하여 다시 입원하여 진료한 결과 골절로 뼈에 핀을 박는 과정에서 과실로 비골신경이 끊어졌다가 다시 이어졌다는 설명을 들을 수 있었다.

2.1 귀하가 판단하는 상기 환자안전 사례의 위험도에 √표시를 해주시기 바랍니다.

____① 근접오류(A) ____② 근접오류(B) ____③ 무해사건(C) ____④ 무해사건(D)

____⑤ 위해사건(E) ____⑥ 위해사건(F) ____⑦ 적신호사건

2.2 귀하가 판단하는 상기 환자안전 사례의 위험도 기간에 √표시를 해주시기 바랍니다.

____① 영구적(Permanent) ____② 일시적(Temporary) ____③ 알 수 없음(Unknown)

[사례 3]

A환자는 입원 20일 전부터 호흡곤란, 부종 등이 있었음을 호소하여 흉부 X-ray검사 등을 시행한 후, 만성심부전(확장성 심근병증으로 인한), 심방세동, 고혈압, 빈혈 등으로 진단하고 진단에 따른 치료를 진행하여 호흡곤란 등의 증세가 호전되어 가던 중 부정맥이 있는 상태로 가슴이 답답함을 호소하였으나 이내 증상은 호전되었다. 퇴원을 앞두고 의료진은 환자의 폐색전증 내지는 심장질환 악화 가능성에 대한 평가를 위해 CT조영술이 필요하다는 설명을 하고, 환자의 동의를 받은 후 CT조영술을 실시하였다. CT조영술 실시 후 환자는 가슴이 답답함을 호소하고, 그 다음날부터는 오심, 구토, 설사 등의 증세를 보이기 시작하였고, 이후 계속 누워자려고만 하는 상태를 보였다. 이에 의료진은 환자의 상태를 조영제에 기인한 급성신손상 및 저나트륨혈증, 코칼륨혈증으로 진단하고 이에 대한 치료를 시행하였다.

3.1 귀하가 판단하는 상기 환자안전 사례의 위험도에 √표시를 해주시기 바랍니다.

____① 근접오류(A) ____② 근접오류(B) ____③ 무해사건(C) ____④ 무해사건(D)

____⑤ 위해사건(E) ____⑥ 위해사건(F) ____⑦ 적신호사건

3.2 귀하가 판단하는 상기 환자안전 사례의 위험도 기간에 √표시를 해주시기 바랍니다.

____① 영구적(Permanent) ____② 일시적(Temporary) ____③ 알 수 없음(Unknown)

[사례 4]

80세의 남자 환자로 홀렙 수술을 받던 중 방광목과 전립선와에 출혈이 발생하여 개복술을 받았다. 수술 후 환자는 심한 복통을 호소하였고, 심박동수가 140/m 이상 상승하여 일반외과 협진 하에 복부 골반 전산화단층촬영을 받았고, 그 결과 소장 천공에 의한 복막염 및 양측성 흉수 소견에 따라 천공된 소장을 절제하는 시험적 개복술을 다시 받았다. 이후 환자에게 고열 등 패혈증 유사 증상이 나타나자, 혈액배양검사를 실시하였고, 그 결과 다제내성균 중 하나인 스타필로코커스 에피데르미데스(Staphylococcus Epidermidis)가 검출되어 반코마이신을 투여하였다. 반코마이신 투여 후 환자에게 혈변 증상이 나타나자 의료진은 신장내과 협진을 통하여 반코마이신 투여에 따른 급성신부전으로 의증하고 반코마이신 투여를 중단하였으며, 지속적 신대체요법(CRRT, Continuous renal replacement therapy)을 실시하였다. 그러나 환자의 혈변이 계속되자 장내시경 및 상부위장관내시경을 시행하였고, 두 번째 수술부위인 소장의 재출혈을 의심하였으며, 소장의 일부를 다시 절제하는 수술을 시행하였다. 세 번째 수술 이후 환자의 산소포화도가 87%까지 감소하자 의료진은 기관내삽관을 시행하였고, 혈액배양균 검사에서 스타필로코커스 및 칸디다가 검출되어 테이코플라닌과 메로페넴 항생제를 투여하였다.

4.1 귀하가 판단하는 상기 환자안전 사례의 위험도에 √표시를 해주시기 바랍니다.

____① 근접오류(A) ____② 근접오류(B) ____③ 무해사건(C) ____④ 무해사건(D)

____⑤ 위해사건(E) ____⑥ 위해사건(F) ____⑦ 적신호사건

4.2 귀하가 판단하는 상기 환자안전 사례의 위험도 기간에 √표시를 해주시기 바랍니다.

____① 영구적(Permanent) ____② 일시적(Temporary) ____③ 알 수 없음(Unknown)

[사례 5]
바쁜 상황에서 검사처방을 한꺼번에 수행하기 위해 A환자 수혈을 위한 혈액을 채취한 후 혈액을 검체용기에 담았으나 사전에 라벨링이 되어 있던 B환자의 검체용기에 담게 되어 B환자를 위한 혈액이 준비되어 있었다. A환자를 위한 혈액 처방이 있어 불출됨에 따라 A환자에게 B환자의 혈액이 수혈되게 되었다. A환자는 수혈이 시작되자 곧바로 가슴이 답답하다고 호소하여 즉시 수혈을 중단하고 확인한 결과 검체가 바뀌었음을 확인하였다.

5.1 귀하가 판단하는 상기 환자안전 사례의 위험도에 √표시를 해주시기 바랍니다.

____① 근접오류(A) ____② 근접오류(B) ____③ 무해사건(C) ____④ 무해사건(D)

____⑤ 위해사건(E) ____⑥ 위해사건(F) ____⑦ 적신호사건

5.2 귀하가 판단하는 상기 환자안전 사례의 위험도 기간에 √표시를 해주시기 바랍니다.

____① 영구적(Permanent) ____② 일시적(Temporary) ____③ 알 수 없음(Unknown)

[사례 6]
환자는 제4번, 제5번 요추의 추간판이 제 자리에서 벗어나 척추신경을 압박하는 증상인 추간판탈출증으로 병원에서 4,5번 척추 사이의 추간판을 원래대로 돌려놓는 수술을 받았다. 수술 당시 의료진은 제4,5번 척추에 의료용 클립을 붙인 다음에 해당 추간판을 절개하는 수술을 진행하였으나, 의료진이 실제 의료용 클립을 부착한 척추는 제 4,5번 척추가 아니라 제 3,4번 척추였음을 나중에 발견하였고 의료진은 해당 상황을 수습하고 수술을 마쳤다. 수술 후 환자는 제 3,4,5번 추간판에서 화농성 염증이 일어나기 시작했고, 제1번 천추와 제5번 요추의 추간판 탈출이 일어나게 되었으며, 이로 인해 척추신경이 눌리면서 결국 성기능 장애까지 이어지게 되었다.

6.1 귀하가 판단하는 상기 환자안전 사례의 위험도에 √표시를 해주시기 바랍니다.

____① 근접오류(A) ____② 근접오류(B) ____③ 무해사건(C) ____④ 무해사건(D)

____⑤ 위해사건(E) ____⑥ 위해사건(F) ____⑦ 적신호사건

6.2 귀하가 판단하는 상기 환자안전 사례의 위험도 기간에 √표시를 해주시기 바랍니다.

____① 영구적(Permanent) ____② 일시적(Temporary) ____③ 알 수 없음(Unknown)

[사례 7]
환자는 폐암진단으로 좌측 폐엽 절제술을 받았으며 수술장에서 흉관을 삽입한 채로 병실로 왔다. 수술 후 3일째 되는 날 담당간호사는 밤번 근무 중 환자의 흉관 배액병에 배액이 많이 찬 것을 보고 배액병을 교환하기로 하였다. 새로운 흉관 배액병과 clamp할 켈리 등을 준비하여 환자에게 간 간호사는 배액병 교환을 위해 환자쪽의 흉관을 켈리로 잠그고 배액병과 흉관이 연결된 부분을 분리하였다. 그 순간 환자가 움직이면서 느슨하게 잠겨 있던 켈리가 풀렸고 환자는 숨을 들이쉬면서 불편감을 호소하였다. 간호사는 그동안 켈리가 풀린 적이 없었기 때문에 켈리가 풀릴거라고는 생각하지 못했다. 간호사는 새로운 배액병에 흉관을 연결하고 환자에게 기침을 시킨 후 담당의에게 보고하였다. 환자는 처음 기침을 할 때는 흉관으로 공기가 나오는게 보였으나 더 이상 나오지 않았고 바로 촬영한 흉부 방사선촬영에서도 별 문제는 없었다.

7.1 귀하가 판단하는 상기 환자안전 사례의 위험도에 √표시를 해주시기 바랍니다.

____① 근접오류(A)　____② 근접오류(B)　____③ 무해사건(C)　____④ 무해사건(D)

____⑤ 위해사건(E)　____⑥ 위해사건(F)　____⑦ 적신호사건

7.2 귀하가 판단하는 상기 환자안전 사례의 위험도 기간에 √표시를 해주시기 바랍니다.

____① 영구적(Permanent)　____② 일시적(Temporary)　____③ 알 수 없음(Unknown)

[사례 8]
응급실에 흉통을 주호소로 내원한 34세 남자 환자에게 간호사는 22시 50분경 isoket 50mg을 수액에 희석하여 수액주입기구를 이용해 10cc/hr로 주입을 시작하였고, 23시 35분경 premedication 후 수액주입기구를 유지한 상태로 chest 3D CT 검사를 위해 CT실로 가서 촬영 후 23시 55분경 바로 일반병실로 입실하였다. 입원 후 담당간호사는 isoket 50mg이 희석된 수액이 full drop되어 150cc가 주입되었음을 발견하였고 즉시 주치의에게 보고하였다. CT실 당직자에게 확인하니 CT 촬영을 위해 수액주입기구를 제거한 상태로 조영제를 주입한 후 수액조절기를 잠갔는지 잘 기억이 나지 않는다고 하였고, 환자를 이송한 요원은 CT실에서 환자가 나온 상태 그대로 환자를 이송하였다고 하였다.

8.1 귀하가 판단하는 상기 환자안전 사례의 위험도에 √표시를 해주시기 바랍니다.

____① 근접오류(A)　____② 근접오류(B)　____③ 무해사건(C)　____④ 무해사건(D)

____⑤ 위해사건(E)　____⑥ 위해사건(F)　____⑦ 적신호사건

8.2 귀하가 판단하는 상기 환자안전 사례의 위험도 기간에 √표시를 해주시기 바랍니다.

____① 영구적(Permanent)　____② 일시적(Temporary)　____③ 알 수 없음(Unknown)

[사례 9]
특발성 폐섬유증 환자의 혈액 균 배양 검사 결과 Yeast가 자란다는 보고에 따라 주치의는 Amphotericin B를 사용하기로 하였으며, 안전을 위해 test dose 1mg을 주입 후 4시간 후에 24mg을 주고 다시 12시간 후에 나머지 25mg을 투여한 후 매일 50mg을 사용하기로 결정하고 Amphotericin B test dose 1mg → 4시간 후 24mg → 16시간 후 25mg으로 투약 처방을 내렸다. 담당간호사 A는 Amphotericin B를 실제로 투약해본 경험이 없어 담당주치의의 지시대로 오후 1시에 test dose 1mg, 오후 5시에 24mg, 오후 5시 기준으로 16시간 이후인 다음날 오전 9시에 25mg을 투약하도록 스케줄링을 하고 초번 간호사에게 인계하였다. 초번 간호사에게 인계받은 밤번 간호사 B는 다음날 처방에 오전 5시에 Amphotericin B 25mg 투여 지시가 있었으나 주치의의 시간계산 착오라고 생각하고 그대로 오전 9시에 투약하도록 낮번 간호사에게 인계하였다. 낮번 간호사가 오전 9시에 투약하기 전에 주치의에게 확인하니 16시간 기준은 test dose가 투여된 오후 1시가 기준이었음을 알게 되었다.

9.1 귀하가 판단하는 상기 환자안전 사례의 위험도에 √표시를 해주시기 바랍니다.

____① 근접오류(A) ____② 근접오류(B) ____③ 무해사건(C) ____④ 무해사건(D)

____⑤ 위해사건(E) ____⑥ 위해사건(F) ____⑦ 적신호사건

9.2 귀하가 판단하는 상기 환자안전 사례의 위험도 기간에 √표시를 해주시기 바랍니다.

____① 영구적(Permanent) ____② 일시적(Temporary) ____③ 알 수 없음(Unknown)

♣ 끝까지 응답해 주셔서 대단히 감사합니다!! ♣
