# Supplementary material for: Differences in the perception of harm assessment among nurses in the patient safety classification system
Source: PLoS One. 2020 Dec 7;15(12):e0243583. doi: 10.1371/journal.pone.0243583 (PMC7721130; doi:10.1371/journal.pone.0243583)
Supplement: Attachment — Submitted filename: Response to reviewers_15Sep2020.docx [file pone.0243583.s004.docx]

Dear Editor and Reviewer:

We would like to thank the reviewers for their thoughtful review on our manuscript entitled “Differences in the perception of harm assessment among nurses in the patient safety classification system”. Those comments are all valuable for revising and improving our paper. We agree with their comments and we have revised our manuscript accordingly.

Please find our responses to the reviewers’ comments below.

***Response to comments from editor and reviewers:***

**Journal Requirements:**

1) Please ensure that your manuX-X-SCRIPT meets PLOS ONE's style requirements, including those for file naming.

**Response:** We have modified our manuscript and file name by referring to the PLOS ONE’s style requirements.

2) In your financial disclosure, please clearly specify whether the funders played any role in the study.

**Response:** We have specified the role of funders in the financial disclosure.

3) Please provide further details regarding how participants were recruited, including the participant recruitment date.

**Response:** We have added how we recruited subjects including the recruitment date. Please refer to ‘Subject of Study’.

4) Please clarify in the methods section where the nurses were recruited from (which hospital/study sites).

**Response:** We have specifically added from which institution the subjects were recruited. Please refer to “Subject of Study”.

5) Thank you for providing the Korean version of the questionnaire as Supporting Information. Please also include an English copy as Supporting Information.

**Response:** We would include an English version of the questionnaire.

6) Please amend your current ethics statement to include the full name of the ethics committee/institutional review board(s) that approved your specific study. Once you have amended this/these statement(s) in the Methods section of the manuX-X-SCRIPT, please add the same text to the “Ethics Statement” field of the submission form (via “Edit Submission”).

**Response:** We added the full name of the ethics committee/institutional review board in the section of “Ethical Considerations” and “Ethics Statement” field of the submission form.

In the case of Yonsei University Wonju Severance Christian Hospital, one of the institutions that conducted the study, only the approval of the nursing department was required for research targeting nurses, so the study was conducted with the approval of the Sangji University IRB, which the principal investigator belongs to. Please refer to “Ethical Considerations”.

**Reviewers' comments:**

1) According to the author, this study is based on the fact that accurate assessment of harm by healthcare providers is highly important, and that factors such as lack of adequate training can lead to differences in perceptions of harm. However, there is no citation from the literature on this in the Introduction section.

**Response:** We have described the differences in perception between system reporters among the factors that have not been reported more accurately by the patient safety incident reporting system, and added to the Introduction section on the need for education. Please refer to “Introduction”.

2) The relationship between the importance of standardization of classifications and terminology in incident reporting, which the author mentions in the Introduction and at the beginning of the Discussion, and the differences in nurses' perceptions of events (particularly in terms of impact on patients and duration of harm) that this study tried to identify is unclear. I think that this lack of clarity can be attributed to the lack of reference to how differences in nurses' perceptions of harm may be detrimental to incident reporting.

**Response:** We have described in the Discussion section how differences in perception among nurses can be a problem for the patient safety reporting system. Please refer to “Discussion”.

3) This problem also affects the Discussion. The first paragraph of the Discussion is what should be stated in the Introduction, and the second paragraph just redefines what has already been defined in the Methods. In the Discussion, starting from the third paragraph, the results of this study, the following sections have not been adequately discussed: 1) General Characteristics of subjects, 2) Subjects' patient safety event experiences, and 3) Consistency Rate among subjects regarding the scenarios. This may be because the purpose of this study is not properly established in the Introduction by citing the literature. Therefore, the authors did not compare the results of this study to the findings of previous studies and show what new findings this study represents in this research area. The paragraph from line 228 suddenly refers to education, but the authors should mention more about the relationship between education and this study in the Introduction.

**Response:** We have revised the Discussion section as a whole with reference to your valuable comments. Please refer to “Discussion”.

4) In summary, it is necessary to clearly state what this study tries to identify in the field of incident reporting and patient safety and to state that this requires a method of determining the rate of agreement between the nurses' perceptions of harm based on the scenarios. The results obtained then need to be discussed in relation to previous studies.

**Response:** We have clearly described what this study seeks to identify in the areas of patient safety reporting system, and stated that a method for determining the rate of agreement between nurses' perceptions of harm is needed. Please refer to “Discussion”.

**Minor comments:**

1) p.7, line 139 - I think the author forgot to delete the phrase, "Contents of a patient safety training."

2) p.7, line 141 - Please make sure the phrase "the number of patients." is correct.

3) p.9, line 157 - Please define "patient safety event."

4) p.9, line 163 - It is difficult to understand the phrase, "medication error was the most common answer ".

5) p.10, line 189 - Please use the word for Fleiss' assessment of the Kappa value in the Method section of line 123. "Showing a certain degree of, but very low, agreement" is what I think should be included in the Discussion part.

6) p.12, line 203 - Please reconsider the transition "therefore"; I think that the relationship between the preceding and following sentences is not cause and effect.

7) p.12, line 213 – Among other things, the author defines near misses here; however, it is unclear how this relates to the definition on p.5, line 98. Also, if you are going to provide a definition, please describe it in the Methods section.

**Response:** As suggested by the reviewer, we have reviewed carefully the entire manuscript and have modified referring to the minor comments.

**Reviewer #2 comments:**

1) Please provide a structured abstract.

**Response:** We have revised the abstract. Please refer to “Abstract”.

2) Please provide the English translation of Korean version of the questionnaire.

**Response:** We would submit an English version of the questionnaire.

3) Please provide more information about the validity of Korean version of the questionnaire. Did you calculated judgmental or empirical validity?? The information about validity should be included completely in the manuX-X-SCRIPT. And if you do not, there is a big flaw in your work.

4) There was a need to follow the questionnaire validity steps in your work so that we can trust the results of your study.

**Response:** We developed the Korean version of the questionnaire to evaluate the reliability of harm score assignment entered into the patient safety incidents reporting system by nursing staff. We prepared the questionnaire by referring to the paper below, and the questionnaire was consisted of nine scenarios of patient safety incidents occurring in the clinical field of Korea and required the subjects to evaluate the harm and harm duration of each scenario. Also, after completing the Korean version of the questionnaire, we received reviews and feedback from experts working at clinical and university on whether the scenario of patient safety incidents in the questionnaire reflects the real-world incidents. We have described these details more clearly in the Research Tools, Instrument section.

- **Williams T, et al. The reliability of AHRQ Common Format Harm Scales in rating patient safety events. J Patient Saf 2015 Mar;11(1):52-9.**

Please refer to the “English version of the questionnaire” & “Instrument”.

5) The questionnaire detail should be completely included in the methods.

**Response:** We have revised the details of questionnaire in Instrument section. Please refer to the “Instrument” & “Definition of Harm and Harm Duration of Questionnaire”.

6) I did not find any information about reliability of the questionnaire.

**Response:** To evaluate the reliability of harm score assignment, we calculated the Fleiss’ kappa which is a statistical measure for assessing agreement among multiple raters assigning categorical ratings. Please refer to the “Data Analysis”.

7) Line 98-99: Classification of harm and harm duration.

**Response:** We described about the classification of harm and harm duration in the Definition of Harm and Harm Duration of Questionnaire section. Please refer to the “Definition of Harm and Harm Duration of Questionnaire”.

8) the item A and B is “near miss”?? Is that correct?

**Response:** The harm assessment criteria used in our study were developed by NCC MERP, which is currently widely applied in harm assessment in Korean medical institutions. A detailed description of near miss A and B is provided in the Definition of Harm and Harm Duration of Questionnaire section. Please refer to the “Definition of Harm and Harm Duration of Questionnaire”

9) You could develop data analysis and perform a regression model between participants characteristics and patient safety event experiences.

**Response:** Thank you for your valuable comment. In this study, we intended to evaluate the consistency of the harm assessment among nurses, and we would like to conduct further research to analyze and compare the general characteristics such as patient safety experience and their relevance to the harm assessment.

10) General Characteristics form should be introduced in the methods.

**Response:** We have added the details of General Characteristics in the Instrument section. Please refer to the “Instrument”.

11) It is recommended to add the mean and SD for quantitative variables in the General Characteristics of the participants.

**Response:** In this study, all of the general characteristics of the subjects were measured as categorical variables, and therefore we calculated the frequency and percent.

12) Line 130-132: there is need to reworded, It is confusing.

**Response:** We have modified the line 130-132.

13) Please summarize key findings of the study In initial part of the discussion.

**Response:** We have modified the Discussion section. Please refer to the “Discussion”.

We sincerely hope the revision will meet with your requirements.
