# Supplementary material for: Differences in the perception of harm assessment among nurses in the patient safety classification system
Source: PLoS One. 2020 Dec 7;15(12):e0243583. doi: 10.1371/journal.pone.0243583 (PMC7721130; doi:10.1371/journal.pone.0243583)
Supplement: Attachment — Submitted filename: Response to reviewers_26Oct2020.docx [file pone.0243583.s005.docx]

Dear Editor and Reviewers:

We would like to thank the reviewers for their thoughtful review on our manuscript entitled “Differences in the perception of harm assessment among nurses in the patient safety classification system”. Those comments are all valuable for revising and improving our paper. We agree with their comments and we have revised our manuscript accordingly.

Please find our responses to the reviewers’ comments below.

***Response to comments from reviewers:***

**Reviewer #1 comments:**

1) Your revision has helped me understand the position of this study in the patient safety field. I think the methods, results, and discussion sections are adequate. However, your conclusions need to be brief and correspond to the introduction section. The conclusions have overlapped slightly with what has been stated in your results and discussion sections. I would suggest that you instead try to write a straightforward description of the most important findings of this study. I understand the background and purpose of this study as follows. For quality reporting of patient safety event, it is important for the reporter to accurately categorize the degree of patients’ harm. In particular, it is important to standardize the assessment of the degree of harm by nurses because they have many opportunities to report it. Although some studies have considered the standardization of the assessment of patients’ harm in other countries, the issue remains unclear in Korea. Therefore, this study aims to determine whether Korean nurses accurately judge the degree of harm by using a scenario to determine the variability in harm ratings. The conclusions for this introduction could be as follows. For quality patient safety incident reporting, it is important for healthcare professionals to accurately assess the degree of patients’ harm. This study found that the assessment of the degree of harm by Korean nurses was not standardized. The reason for this variability could be due to the lack of education that takes harm assessment into account. Therefore, training in harm assessment and the development of programs to support this training are both necessary.

**Response:** Thank you for your valuable and kind comments. We have revised the Conclusion section more clearly with reference to your comments. Please refer to “Conclusion”.

**Reviewer #2 comments:**

1) Line 11: Should be “tool”. In this study for assessing the validity of the developed tool only content validity with 3 experts (without pilot study, face validity, criterion validity) was used. This issue should be mentioned as a main limitation.

**Response:** We described about the main limitation on the validity of the questionnaire with reference to your valuable comments in the Discussion section. Please refer to the “Discussion”.

We sincerely hope the revision will meet with your requirements.
