# Supplementary material for: Differences in the perception of harm assessment among nurses in the patient safety classification system
Source: PLoS One. 2020 Dec 7;15(12):e0243583. doi: 10.1371/journal.pone.0243583 (PMC7721130; doi:10.1371/journal.pone.0243583)
Supplement: Attachment — Submitted filename: Response to reviewers_20Nov2020.docx [file pone.0243583.s006.docx]

Dear Editor and Reviewers:

We would like to thank the reviewers for their thoughtful review on our manuscript entitled “Differences in the perception of harm assessment among nurses in the patient safety classification system”. Those comments are all valuable for revising and improving our paper. We agree with their comments and we have revised our manuscript accordingly.

Please find our responses to the reviewers’ comments below.

***Response to comments from reviewers:***

**Reviewer #1 comments:**

1) Thank you for revising your conclusions. I now have a clearer understanding of the conclusions of your study. Unfortunately, the conclusions have not been changed in the Abstract, even though the Conclusions have been revised. Could you please consider whether it is necessary to include the revised conclusion in the Abstract? I would appreciate it if you could revise it.

**Response:** Thank you for your comments. We should have revised the abstract as well as revised the conclusion. Unfortunately, we missed that point. We have revised the Abstract section with reference to your comments. Please refer to “Abstract”.

We sincerely hope the revision will meet with your requirements.
